# Identifying the Best Times for Cognitive Functioning Using New Methods: Matching University Times to Undergraduate Chronotypes

**DOI:** 10.3389/fnhum.2017.00188

**Published:** 2017-04-19

**Authors:** M. D. R. Evans, Paul Kelley, Jonathan Kelley

**Affiliations:** ^1^Sociology and Applied Statistics Program, University of NevadaReno, NV, USA; ^2^Sleep, Circadian and Memory Neuroscience, Learning and Teaching Innovation, The Open UniversityMilton Keynes, UK

**Keywords:** later start times, sleep, sleep deprivation, circadian, SCN, pRGC, wake maintenance zone, Geophysical Biological Time

## Abstract

University days generally start at fixed times in the morning, often early morning, without regard to optimal functioning times for students with different chronotypes. Research has shown that later starting times are crucial to high school students' sleep, health, and performance. Shifting the focus to university, this study used two new approaches to determine ranges of start times that optimize cognitive functioning for undergraduates. The first is a survey-based, empirical model (SM), and the second a neuroscience-based, theoretical model (NM). The SM focused on students' self-reported chronotype and times they feel at their best. Using this approach, data from 190 mostly first and second year university students were collected and analyzed to determine optimal times when cognitive performance can be expected to be at its peak. The NM synthesized research in sleep, circadian neuroscience, sleep deprivation's impact on cognition, and practical considerations to create a generalized solution to determine the best learning hours. Strikingly the SM and NM results align with each other and confirm other recent research in indicating later start times. They add several important points: (1) They extend our understanding by showing that much later starting times (after 11 a.m. or 12 noon) are optimal; (2) Every single start time disadvantages one or more chronotypes; and (3) The best practical model may involve three alternative starting times with one afternoon shared session. The implications are briefly considered.

## Introduction

Education and work generally start at fixed times, mostly early and with no adjustment for different chronotypes among those who study and work. However, in adolescence and early adulthood optimal wake and sleep times are shifted 2–3 h later in the day, and yet this group are still required to conform to education start times more appropriate to young children and older adults. Traditionally, institutions have tried to tailor the humans to the organization, but research suggests that, at least as far as time is concerned, it is more efficient, productive, and humane to align the organization's schedules to the natural time patterns of the humans who study and work in them.

Despite an impressive, cumulating body of medical and educational research evidence consistently indicating that later start times improved educational performance (Borlase et al., 2013; Edwards, 2012; Meltzer et al., 2014; Wahlstrom et al., 2014), there has been little change in educational starting times. Even Korea's Gyeonggi Province and some school districts in the United States such as Seattle that have made big changes generally have starting times no later than 09:00. Perhaps more worrying, there is little research to illuminate what starting times would be optimal in education, particularly for universities.

The crux of the matter in the temporal misalignment problem is that biological changes beginning in puberty shift wake and sleep times 2–3 h later in the day. This shift is at its greatest at age 19 (Roenneberg et al., 2004) before reverting to an earlier pattern in the mid-20s. Oblivious to these changes, secondary schools and universities continue to start classes early in the morning.

Genetic factors lead to variations in circadian timing of ±4 h from the mean, as well as differences by age and by sex. For instance, the shift in adolescent circadian timings to 2–3 h later begins earlier in females and reaches its peak at 19.5 years, whereas it is 20.9 in males (Roenneberg et al., 2004). In extended family groups the variations of ±4 h from the mean combined with the time shift in adolescence ensures that during 8 h sleep for most of the group there would be family members naturally alert. In evolution humans tended to live in extended family groups of <150—a maximum size for bilateral relationships of obligation and reciprocity (Dunbar, 2014). It is likely that this 24-h protection during sleep may have had many advantages. In modern times this degree of variation suggests that even in relatively small groups a single starting time for education is unlikely to be optimal for all individuals.

The temporal misalignment between the sleep timing shift and educational institutions' usual hours causes significant sleep loss. Sleep loss, in turn, impairs academic performance and also elevates risks of obesity, depression, and drug abuse. The biological mechanisms through which early starts increase health risks and lower performance are well-established (Carrell et al., 2011; Kirkby et al., 2011; Kelley et al., 2015).

Early education start times for students in the 14–24 age range are linked to chronic, irrecoverable sleep loss of more than 2 h each day (Foster et al., 2013; Kelley et al., 2015). Because these changes in circadian timing conflict with early starts in school and university, sleep deprivation increases rapidly with age (Roenneberg et al., 2007). More generally, prior research shows that sleep deprivation damages physical and emotional well-being and impairs cognition and performance (Lockley et al., 2004; Blakemore and Choudhury, 2006). Sleep loss or mistimed sleep are associated with increased risk of metabolic disorders, obesity, and diabetes (Buxton et al., 2012; Luyster et al., 2012); depression, anxiety, and drug use (Preckel et al., 2013); and poorer attention, performance, and memory consolidation (Goldstein et al., 2007).

A possible solution lies in later start times, as recommended by the Centers for Disease Control and Prevention, the American Academy of Pediatrics, and the American Medical Association (American Academy of Pediatrics, 2014; Wheaton et al., 2015): middle and high schools should open no earlier than 08:30. This early time reflects the limitations of almost all previous research to starting times no later than 09:00. Nevertheless, the evidence supporting later start times, largely medical and sleep-research based, show that later start times would reduce health risks for adolescents (Hansen et al., 2005; Millman, 2005; Sawyer et al., 2012; El-Sheikh et al., 2013; Basch et al., 2014; de Souza and Hidalgo, 2014).

Compared to the growing body of evidence on secondary schools, studies of university start times are relatively rare despite the demonstrated importance of later times for optimal academic performance (Matchock and Mordkoff, 2009; Carrell et al., 2011; Hsu et al., 2012). Findings to date reveal that undergraduates also have working hours that begin too early and so incur the same risks as high school students. The biological mechanisms for these risks can be remarkably rapid. In a single week with <6 h sleep, subjects aged 27.5 ± 4.3 y showed changes in metabolic, immune, inflammatory and stress responses, gene expression, alertness and performance (Möller-Levet et al., 2013). Sleep disruption also can impair specific cognitive functions such as working memory (Lo et al., 2012). A recent functional magnetic resonance imaging (fMRI) study (Muto et al., 2016) demonstrated that cortical responses showed significant circadian rhythmicity, the phase of which varied across brain regions. Moreover, subjects (17 men, 16 women; aged 21.1 ± 1.7 y) showed local modulation of cerebral circadian phase in cognitive functions in responses to task-related requirements such as attention and working memory. One aspect of these experiments suggested increased cortical responses sometimes occurred during the period before onset of melatonin secretions, or the wake maintenance zone (WMZ). In all, their findings suggest local, region-specific, task-dependent circadian influence on cortical functions.

In order to identify timing ranges that elicit peak performance in university students, and to create a generalizable method for determining suitable timing ranges in other contexts, we used two new approaches: a survey-based, empirical model (SM), and a neuroscience-based, theoretical model (NM). After data analysis, these different approaches independently identified suitable—and highly similar—timing ranges.

## Method: survey-based empirical model

The survey-based, empirical model (SM) was created based on previous research models created by the International Survey Center, a non-profit scientific organization specializing in survey design and analysis (International Survey Center, 2017; for examples of prior surveys see Evans and Kelley, 2011, 2014). The subjects analyzed here were 190 mostly first and second year university students from a large North American public university pursuing a range of degree programmes as part of the general quantitative analysis programme of study required by the university.

Subjects were asked a series of questions including a self-assessment of their own chronotype, preferred sleeping times, and a variety of other time-related matters. In an important extension to prior research, subjects were also asked to rate their fitness for cognitive activities in each hour of the 24-h day. Prior research on survey design shows that especially during exploratory research very detailed questions about time-related actions and feelings produce much more reliable data than do summary questions (Tourangeau et al., 2000; Schaeffer and Presser, 2003). The survey analysis created a full 24-h circadian profile for each student and also collected information on their self-identified chronotype.

The key series of survey questions began:

Do you usually FEEL AT YOUR BEST at these times…

**Table:** 

	Yes!!	yes	??	no	No!!
5 a.m.	◦	◦	◦	◦	◦
6 a.m.	◦	◦	◦	◦	◦

and continued hour-by-hour through 4 a.m. the next morning. Answers were scored conventionally in equal intervals, for clarity and without loss of generality as points out of 100: Yes!! = 100 points; yes = 75; ?? = 50; no = 25; No!! = 0. The equal interval assumption is potentially problematic but scoring by ordinal probit methods which eschew that assumption leads to virtually identical results (Kelley et al., 2010, Technical Appendix in Supplementary Material).

Statistical analysis used a range of standard techniques, for example Table 1 gives the means and the standard deviations, and significance tests for the difference of each standard deviation from the average standard deviation (statistically significant differences are marked with an asterisk). On the basis of chance alone, there should be approximately 1 significant difference out of the 24 (at *p* = 0.05), but there are, as shown below, 7.

**Table 1:** **Questions “Do you usually feel at your best at these times…” asked for each hour of the day and scored: definitely yes = 100; yes = 75; Mixed, undecided = 50; no = 25; and definitely no = 0**.

**Variable**	**Mean**	**Std. Dev**.	**Cases**
5 a.m.	18.8	24.6	190
6 a.m.	20.5	24.7	189
7 a.m.	26.6	28.5	187
8 a.m.	36.6	31.8^*^	192
**9 a.m**.	**50.9**	31.8^*^	**192**
**10 a.m**.	**63.3**	29.0^*^	**194**
**11 a.m**.	**69.1**	**25.4**	**191**
**12 noon**	**71.7**	**24.9**	**191**
**1 p.m**.	**73.2**	**24.1**	**191**
**2 p.m**.	**71.5**	**24.1**	**191**
**3 p.m**.	**70.6**	**24.9**	**189**
**4 p.m**.	**70.9**	**25.0**	**191**
**5 p.m**.	**71.3**	**24.5**	**190**
6 p.m.	71.6	23.5	191
7 p.m.	74.0	22.8	191
8 p.m.	73.9	23.0	190
9 p.m.	73.0	24.2	189
10 p.m.	67.2	27.6	190
11 p.m.	55.6	29.5^*^	188
12 midnight	46.5	30.0^*^	188
1 a.m.	36.8	29.3^*^	188
2 a.m.	30.2	28.0	188
3 a.m.	22.5	23.7	189
4 a.m.	21.0	23.9	189

**Figure 1:**
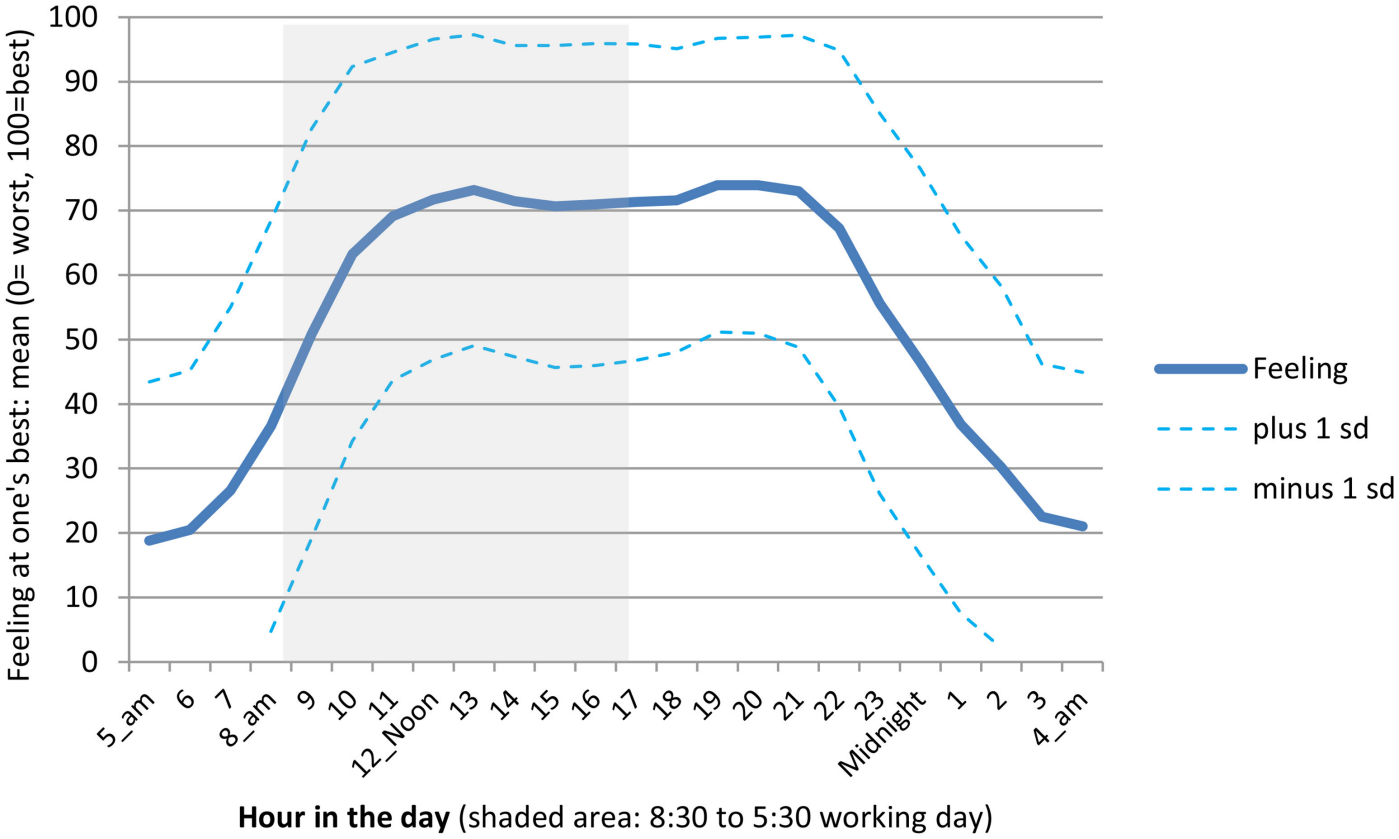
**Time of day 1 feels at their best: 5 a.m.? 6 a.m.? …etc…(24 separate questions)**. Mean ± one standard deviation (standard error of the mean is around 2 points). US undergraduates; N approximately 190, varying slightly by question.

*Means and standard deviations. US undergraduates. See Figure 1. Nine to five working day highlighted in bold*.

* *Significantly different from the average of the standard deviations at p < 0.05 (Chi-square test)*.

The means and the range (±1 standard deviation) for each hour of the day for the sample as a whole are given in Figure 1. The average rating across the 24 h is 53.6. The mean for each of the 24 h is significantly different from this average (*p* < 0.05) except for 9 a.m. and 11 p.m.

This seems tap much the same “optimal time of day concept” as an alternative series of questions: “How awake and alert do you feel…Very alert!/Alert/Somewhat alert/In between/Somewhat sleepy/Sleepy/Very sleepy!” Answers to the two questions are highly correlated (*r* = 0.58 on average) and they show the same time patterns (see Technical Appendix in Supplementary Material: Further details on the survey calculations).

Self-described chronotype was ascertained by a single question:

Do you consider yourself a morning person or an evening person?

◦ Definitely morning!!
◦ Probably morning
◦ Mixed, unsure
◦ Probably evening
◦ Definitely evening!!

About a quarter of the students chose definitely or probably morning, another quarter were mixed or unsure, a quarter probably evening, and another quarter definitely evening. So there was a predominance of later “owl” chronotypes.

**Figure 2:**
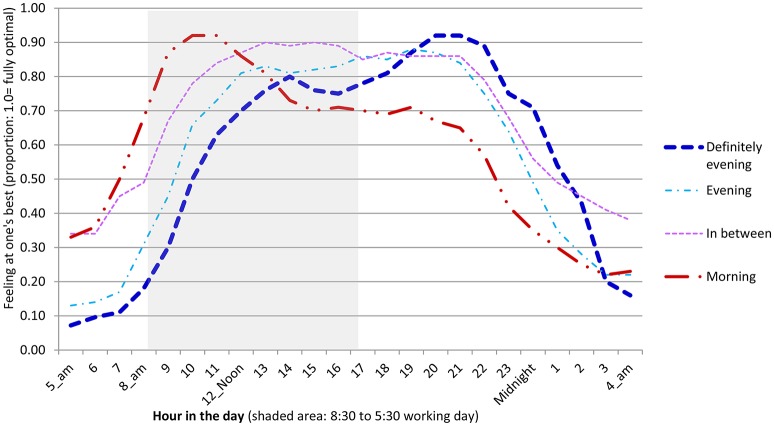
**Personally optimal time of day by self-assessed chronotype**. US university students. N approximately 190 varying slightly from question to question, about equally divided into morning, mixed, evening, and “definitely” evening chronotypes. Means.

**Figure 3:**
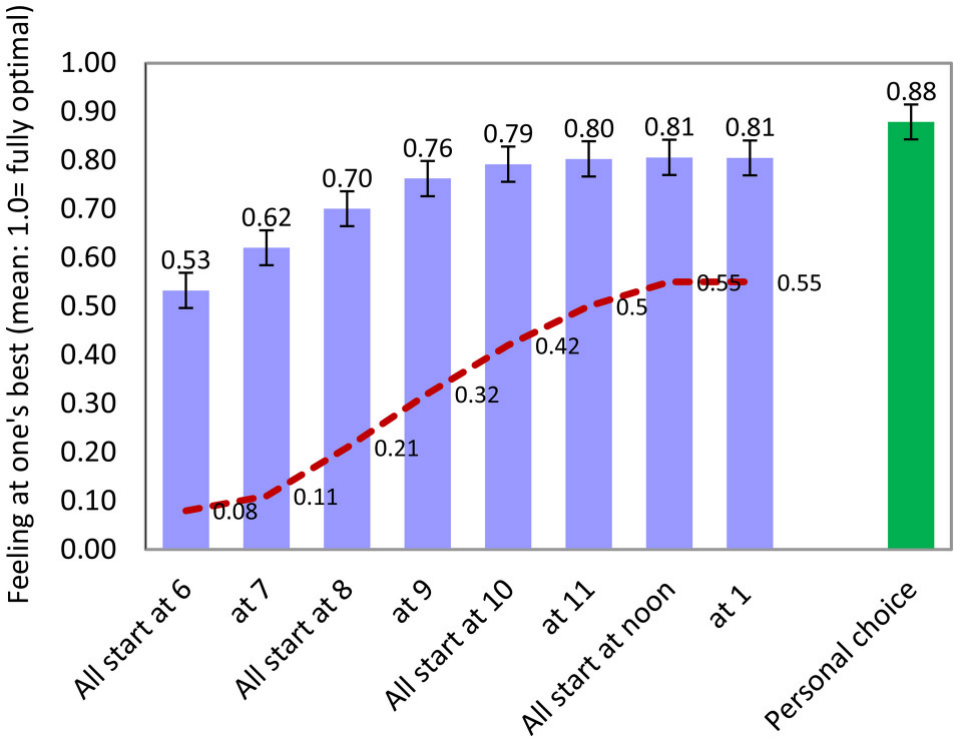
**Optimality of various start times in a 6-h work day: Means**. N about 190 each. Personal choice of start (right, green) is significantly better (*p* < 0.001). Dashed line: 1st hour in block is one of respondent's personal optimums (proportion).

The statistical methods used to analyze these data include descriptive statistics (frequency distributions, means, and standard deviations for the whole sample and for self-defined chronotype subgroups in Table 1, Figures 1, 2, Appendix Tables A,B in Supplementary Material; Pearson correlations in Technical Appendix Table C of Supplementary Material; alpha scale reliabilities in Technical Appendix, Section 2 of Supplementary Material, text), inferential statistics (significance tests and confidence intervals for inequality of means, inequality of standard deviations in Table 1, Figure 3; regression analysis of optimality at different hours on chronotype in Table 2; factor analysis of hourly optimality in Technical Appendix, Table D of Supplementary Material); simulations combining the results of the foregoing analyses (Table 3, Figure 3).

**Table 2:** **Personally optimal time of day by self-assessed chronotype: OLS regression analysis**.

**PANEL 1: MORNING HOURS**						
	**7 a.m**.	**8 a.m**.	**9 a.m**.	**10 a.m**.	**11 a.m**.	**12 noon**
**Chronotype**	**(1)**	**(2)**	**(3)**	**(4)**	**(5)**	**(6)**
Definitely evening	−37.22^***^	−46.17^***^	−53.34^***^	−39.65^***^	−27.66^***^	−15.63^**^
Evening	−34.18^***^	−38.02^***^	−44.38^***^	−29.54^***^	−22.58^***^	−10.11^*^
Mixed	−12.70^*^	−24.61^***^	−28.57^***^	−21.34^***^	−17.12^***^	−8.232
Intercept: Morning	47.22^***^	63.02^***^	82.14^***^	86.46^***^	86.17^***^	80.85^***^
R-sq	0.295	0.316	0.421	0.262	0.173	0.052
N	178	182	184	185	182	182
**PANEL 2: AFTERNOON HOURS**						
	**1 p.m**.	**2 p.m**.	**3 p.m**.	**4 p.m**.	**5 p.m**.	**6 p.m**.
Definitely evening	−5.508	6.383	4.251	2.979	7.376	11.73^*^
Evening	−2.660	2.660	4.972	6.383	10.11^*^	10.11^*^
Mixed	−0.482	5.220	9.291	7.979	5.471	8.853
Intercept: Morning	76.06^***^	68.62^***^	66.30^***^	67.02^***^	65.96^***^	64.36^***^
R-sq	0.008	0.011	0.017	0.015	0.024	0.039
N	182	183	180	182	181	182
**PANEL 3: EVENING HOURS**						
	**7 p.m**.	**8 p.m**.	**9 p.m**.	**10 p.m**.	**11 p.m**.	**12 midnight**
Definitely evening	15.56^***^	23.88^***^	24.70^***^	29.89^***^	30.21^***^	33.50^***^
Evening	11.17^*^	14.89^***^	12.77^**^	12.15^*^	16.18^**^	9.944
Mixed	6.066	9.195^*^	10.78^*^	11.67^*^	16.87^**^	12.51^*^
Intercept: Morning	65.96^***^	62.23^***^	61.17^***^	53.80^***^	39.67^***^	32.61^***^
R-sq	0.066	0.146	0.134	0.149	0.128	0.164
N	182	181	181	181	179	179

*Optimality is measured by a series of questions “At what time of day do you feel at your best?” asked for each hour of the day and scored: definitely yes = 100; yes = 75; Mixed, undecided = 50; no = 25; and definitely no = 0. Metric regression coefficients*.

* *p < 0.05*,

** *p < 0.01*,

*** *p < 0.001*.

*US university students about equally divided into morning (the reference group), mixed, evening, and “definitely evening” chronotypes*.

**Table 3:** **Selected examples of individual responses: Personally optimal times of day and self-assessed chronotype**.

	**7 a.m**.	**8 a.m**.	**9 a.m**.	**10 a.m**.	**11 a.m**.	**noon**	**1 p.m**.	**2 p.m**.	**3 p.m**.	**4 p.m**.	**5 p.m**.	**6 p.m**.	**7 p.m**.	**8 p.m**.	**9 p.m**.	**10 p.m**.	**11 p.m**.	**12 midnnight**
**1. MORNING CHRONOTYPE**																		
1.	50	75	100	100	100	100	100	75	75	75	50	25	25	25	25	25	25	0
2.	50	75	100	100	100	75	75	75	75	75	50	50	50	50	75	50	25	0
3.	25	25	50	75	75	75	50	50	50	50	50	50	50	50	25	25	25	25
**2. IN BETWEEN**																		
4.	0	0	25	50	75	100	100	100	100	100	75	75	50	50	75	75	75	50
5.	25	25	50	50	75	75	50	50	50	50	50	50	50	50	50	50	50	50
6.	25	25	25	100	100	100	100	100	100	100	100	100	100	50	50	50	25	25
**3. EVENING CHRONOTYPE**																		
7.	0	25	25	50	75	75	75	75	75	100	100	100	100	100	100	100	75	75
8.	0	25	50	75	75	75	75	75	100	100	100	75	75	50	50	50	25	25
9.	.	50	50	50	0	0	100	50	50	75	75	75	0	75	75	75	25	25
**4. DEFINITELY EVENING**																		
10.	0	25	50	75	75	75	75	75	75	100	100	100	100	100	100	100	75	75
11.	0	25	25	50	50	50	50	50	50	50	50	75	100	100	100	100	100	100
12.	0	0	0	0	50	50	50	50	50	50	50	50	50	75	75	75′	50	50
**5. OPTIMALITY ADJUSTMENT FOR CASE #12**^a^																		
Original	0	0	0	0	50	50	50	50	50	50	50	50	50	75	75	75′	50	50
Adjusted	0	0	0	0	67	67	67	67	67	67	67	67	67	100	100	100′	67	67
**6. SOME ATYPICAL PATTERNS**																		
13.	0	0	75	75	75	75	25	25	25	25	25	25	75	75	75	75	75	75
14.	25	75	.	75	100	100	75	75	100	75	75	75	75	100	75	75	75	25
15.	0	0	0	0	25	25	50	75	75	50	50	50	75	100	100	25	25	25

*Optimality is measured by a series of questions “At what time of day do you feel at your best?” asked for each hour of the day and scored: definitely yes = 100; yes = 75; mixed, undecided = 50; no = 25; and definitely no = 0. Personally optimal times are highlighted. US university students*.

a *Adjustment so that each case has the same maximum and so weighs equally in the optimality calculations. The ajustment for each score is: NewScore = OriginalScore/OriginalMaximum. For case #12 that comes to: NewScore = OriginalScore/75. Most other adjusted cases are the same although a few = OriginalScore/50. The decimal point is adjusted to run from 0 to 100 for this table (so it is comparable with raw scores)*.

## Method: neuroscience-based theoretical model

The neuroscience-based theoretical model (NM) was created using relevant circadian and sleep deprivation research. Circadian 24-h cellular mechanisms are genetic, evolutionarily conserved, and found across all photosensitive forms of life (Bass and Lazar, 2016). In mammals there is an additional regulatory mechanism. Although, cells have a 24-h rhythm, they are synchronized by the Suprachiasmatic Nuclei (SCN; Young, 2000). The SCN itself is synchronized to the variations in sunlight when blue light of ~480 nm wavelength enters the eye and strikes photosensitive retinal ganglion cells (pRGC) (Foster and Hankins, 2007). Changes in the light levels in twilight (before dawn or after sunset) entrain circadian timing to the 24 h day. These environmental cues regulate the SCN, creating an automatic, unconscious circadian system. Clock timing based on Coordinated Universal Time (UTC) can vary from this circadian system by an hour or more because of time zones, daylight savings time (DST), traveling to another time zone and other factors. These UTC time variations from environmental cues must be eliminated when determining optimal timings, for example daylight saving time (Medina et al., 2015).

The NM led to creating a novel time scale for this 24-h circadian system: Geophysical Biological Time (GBT). GBT has only two variables: the environmental cues of changing light patterns in each specific geophysical location, and the biological circadian rhythms generated genetically in a species, group, or individuals. Universal Coordinated Time (UCT) is a time scale that deviates from GBT because it is structured on artificial geographic divisions (time zones), creates a single time across these zones that does not reflect changes in light levels, and may have Daylight Savings Time (Medina et al., 2015).

In this study the university's geophysical location only required a small adjustment for GBT of −3 min (as used in the calculation of starting times) assuming a −63 min adjustment during daylight savings time.

When there is circadian synchrony, as when UTC matches individual GBT, humans function well. When there is desynchrony it leads to pathologies and dysfunction, including poorer cognitive function. The genetic mechanisms for circadian time systems and for circadian disorders are now largely understood (Jones et al., 2013; Bass and Lazar, 2016). There are variations in individual GBT of 4 h or more above or below the mean in large groups (<150). In modern times this degree of variation implies that even in relatively small groups as in the 190 subjects in this study a single starting time for education based on a mean is unlikely to be optimal for all individuals.

The circadian time pattern used as a mean GBT was 8 h sleep duration, with spontaneous wake at 08:00 and sleep onset at midnight (derived from Roenneberg et al., 2007). However, those aged 19–20 have much later spontaneous wake times by ~90 min (Roenneberg et al., 2004, 2007; Foster et al., 2013; Kelley et al., 2015), suggesting a mean wake time of 9:30. As well as variation by age, GBT varies by sex: the shift in adolescent circadian timings to 2–3 h later begins earlier in females and reaches its peak at 19.5 years, whereas it peaks at 20.9 in males (Roenneberg et al., 2004). In this theoretical model the working day assumes 2 h were required from wake to prepare and travel to the campus, the university had a 6 h taught day, and 2 h were required to travel and preparation for sleep, a total of 10 h. The meant GBT adjusted for age has 16 waking hours, leaving 6 h of free time. Therefore, there is a wide range of possible starting times within the 16 h of wake. Specific starting times will not suit all student's optimal individual GBT (*i*GBT) preferences. This theoretical model is summarized in Table F of the Technical Appendix in Supplementary Material that includes details of the start and end time calculations, UTC, and GBT times, and worked individual examples.

## Results: start and end times

The neuroscience-based, theoretical model (NM) was used to determine optimal start and end times for students (mean age 19) at the university's geographic location. Assumptions were made that a continuous 6-h duration of work was required, and 2 h of domestic/travel activities after wake and before sleep. There are a range of reasonable options for the working period. The earliest had a start time of 11:27 a.m. and end time of 5:27 p.m. The latest had a start time of 3:27 p.m. and end time of 9:27 p.m. All of these are of course far later in the day than is usual in universities.

The NM model has the clear aim of identifying optimal biological times, thus protecting against sleep loss and circadian disruption insofar as is possible. The model's calculations and potential loss of sleep associated with different starting times are similar to those in the SM findings (see Technical Appendix, Table F in Supplementary Material). Interestingly the use of GMT and UTC has allowed us to quantify circadian desynchrony more precisely. This in turn permits a definition of circadian synchrony for an individual as “when iGBT equals UTC,” and for a group “when mean GMT equals UTC.” The WMZ research would strongly suggest the optimal time for extended cognitive performance would be in the late afternoon and early evening (Shekleton et al., 2013; Muto et al., 2016). There appear to be clear specific links between detailed findings from the NM model and SM models.

The survey-based, empirical model (SM) allowed us to obtain a full 24-h circadian profile for each student, as well as their self-identified chronotype.

In general, students do not feel at their best in the early morning hours. On a scale of 0–100, the average student rated 5 a.m. very low, just under 20 points out of 100 (see Figure 1). This is the nadir of the day. The standard deviation is very wide, but even students whose ratings are a full standard deviation above the group as a whole only rate 5 a.m. in the low 40s. Ratings then rise as the morning wears on, but by the start of many typical working days (8:30 a.m.), the average rating is only about 40 points out of 100, well below more optimal start times with ratings just over 70.

As the clock runs forward, students move toward their peak. They reach a neutral point around 9 a.m., and then begin to move into the positive performance zone. At around 11 a.m. students reach the beginning of a long slightly irregular optimal performance plateau which elicits mean ratings between about 70 and about 74. The plateau comes to an end between 9 p.m. and 10 p.m. perhaps reflecting the WMZ with a steep decline thereafter. Ratings drop to the neutral point of 50 around 11 p.m. down into the 30s by about midnight, and down into the 20s around 2 a.m.

These means reveal several important points about when students feel “at their best”:

1. The peak performance spell starts around 11 a.m. or 12 noon. This is much later than the beginning of the standard workday. This is also much later than many undergraduate classes start.
2. The peak performance spell (the high plateau in the graph) is quite long and extends well into the evening—much later than classes typically run.
3. An irregularity in the long plateau is the slight “two-humped” shape, or bimodal distribution. Such a distribution typically indicates that there are at least two groups with different peaks in the data, an issue to which we return, below, when we look at subjective optimality over the course of the day for subjects with different chronotypes. It does *not* reflect bi-phasic optima for individuals, although it does not rule them out.
4. A usual 9 to 5 workday (shaded area in the graph) begins far too early to be optimal for students, starting when most of them are feeling far from their best. Starting two, three, or even 4 h later would be much better at the beginning of the work day and still come to an end well within the long peak performance plateau.

But time is not a “one size fits all” phenomenon: Students' self-ratings of performance are diverse at all times of day. Yet the degree of dispersion is not uniform. The dispersion of performance ratings is narrowest—25 points out of 100 or less -during the long high plateau of peak performance from about 11 a.m. till about 9:30 p.m. The dispersion is at its widest—28 points out of 100 or more—in the fairly early morning, 7 a.m. till about 10 a.m. and then again late at night, about 11 p.m. till 2 a.m.

## How optimal performance hours vary by chronotype

Students were invited to assess their own chronotype, describing themselves as “definitely morning,” persons, “morning” persons, “in between,” “evening” persons, or “definitely evening” persons. In these data for undergraduates, roughly one quarter are larks, seeing themselves either as “definitely morning” or just “morning” people, one quarter see themselves as “in between,” one quarter see themselves as “evening” persons and another quarter see themselves a “definitely evening” persons. Thus, the full spectrum of chronotypes is represented in these late adolescents/young adults, but the owls (combining “evening” and “definitely evening”) outnumber the larks (combining “morning” and “definitely morning”) about 2 to 1.

Their self-reported chronotype aligns well with the times of day when they report feeling at their best (Figure 2). Even the morning-loving larks get off to a slow start, rating the potential quality of their performance in the low to middle 30s, on average, for 5 a.m. and 6 a.m. Then their performance begins to rise steeply, reaching half optimal functionality (50%) by 7 a.m., 68% functionality by 8 a.m., and move into their optimal zone with functionality over 80% by a bit before 9 a.m. Their peak functionality (average ratings in the high 80s to low 90s) is in a spell from 9 a.m. through noon. It begins to decline, falling to 81% by 1 p.m. and holds at a fairly high plateau around 70% until beginning to decline again around 8 p.m. Larks are pretty well aligned with a standard workday of 8:30 a.m. to 5:30 p.m.: Their entire peak functionality spell is inside the standard day and at no point in the day is their functionality below about 70%.

The self-identified “in between” group also finds the early morning hours a struggle, experiencing <50% functionality through 8 a.m. They move into their peak functionality spell a bit after 10 a.m. Their functionality holds in the middle 80s to low 90s for a long time, till a bit after 9 p.m. and declines thereafter. The standard workday captures less of the “in betweens” peak performance period than for the larks: about 50% of the “in betweens” peak performance period vs. 100% for the larks.

Students with a mildly evening chronotype have a substantially harder time with the early morning hours. They are less than half way to being at their best at any time before 9:30 a.m. Their functionality appears to be a bit lower than for the other groups, so it never reaches 0.9, but the peak is in the middle to high 80s from about 5 p.m. to about 9 p.m. The standard workday of 8:30 a.m. to 5:30 p.m. would capture only about one half hour of their performance peak.

The “definitely evening” chronotype students experience a slightly slower start to their day, not reaching 50% functionality till 10 a.m. Their performance reaches about 80% by 5 p.m. and their performance peak reaches from about 6:30 p.m. to about 10:30 p.m. The standard workday of 8:30 a.m. to 5:30 p.m. would fail to capture any portion of their peak performance spell. The slight, albeit intriguing, drop between 2 p.m. and 4 p.m. is not statistically significant (*t* = 1.6772, n.s. even without a Bonferroni type correction).

All these differences between chronotype are clear and statistically significant for the morning hours (Table 2, Panel 1). During the afternoon there are only a few statistically significant differences (Table 2, Panel 2). The differences are again clear and statistically significant in the night time hours up to midnight (Panel 3).

These results make it clear that the optimal timing of the working day would vary greatly according to chronotype.

Nonetheless, certain university activities might require a single start time. The working day that would be both fairest and most efficient would have high average performance and the minimum performance gap between the morning chronotypes and the “definitely evening” chronotypes. The gap is at its largest—over 40 percentage points—between about 7:30 a.m. and 10:30 a.m., so including these hours in a workday for all would be very wasteful of potential functionality. The smallest performance gaps (absolute value of 5% or less) all fall into the time period: 1 p.m. to about 4:30 p.m.

## Results: optimal start times for a 6-h working day

The results thus far reflect the ratings that students on average gave to their performance at different hours of the day, but if we are to discover the best possible university start times for students, we need also to know their best times for a block of hours covering the entire working day. We assume a 6-h class day which might start alternatively at 6 a.m., or at 7 a.m., or at 8 a.m., and so on up to a 1 p.m. start. For each hypothetical start time, we calculate how well it suits each student.

To clarify the matter, here are some actual examples of students' responses (Table 3).

An example may help clarify the procedure. The first case (Table 3, Panel 1, row 1)—let us call her Nancy—who dislikes very early morning hours. She answered the question “At what time of day do you usually feel at your best?” for 7 a.m. with “mixed, undecided.” That gets a score of 50 (the options and scoring were: Definitely yes = 100; Yes = 75; Mixed, undecided = 50; No = 25; and Definitely no = 0). Nancy likes 8 a.m. better (Yes = 75) and is quite happy with later starts throughout the morning, rating each of these times as fully optimal, could not be better (Definitely yes = 100). Thus, Nancy would find the 7 a.m. start to the work day a little below her optimum: 50 + 75 for the first 2 h and 100 + 100 + 100 + 100 for the next four, an average of 88 for all 6 together. That is 0.88 (or 88%) of what would be the best possible work day for her, be a full 100 for each of the 6 h. Nancy's is an unusually favorable rating for a 7 a.m. start (Figure 2, second bar from the left, shows that is only 62% optimal for the average student).

Starting at 8 a.m. would take Nancy even closer to her optimum: (an average of 96% of optimal). A 9 a.m. start would be just as good. But later starts are progressively worse, as the end of the work day goes deeper into Nancy's not-so-favorite afternoon hours. For example Nancy would rate a 2 p.m. start only 54% optimal. Evening starts would be dreadful for early-bird Nancy.

Other students would of course have different preferences than our Nancy. For example the definite “owl” in Panel 4 line 10 would hate anything in the morning but be delighted with a 4 or 5 p.m. start. The Technical Appendix in Supplementary Material provides details on the calculations. One complication is that, as usual with survey measures, a few students rate no particular time as fully optimal (van Vaerenbergh and Thomas, 2013). See the examples in Table 3 Panel 5, and details on the adjustment procedure for those cases in the table's footnote. Note that it is plausible to think in terms of optimal blocks of time, because ratings of adjacent hours are highly correlated (Technical Appendix, Table C in Supplementary Material) and even to cohere into 3 main blocks of hours roughly corresponding to morning, afternoon and evening (factor analysis in Technical Appendix, Table D of Supplementary Material).

All in all, Figure 3 (leftmost bar) gives the rating for a 6 a.m. start averaged over all students in our sample—this is our estimate for a “one size fits all” 6 a.m. university start. The next bar gives our estimate for 7 a.m. university start for everyone, and so forth up to a 1 p.m. start time.

Early starts around 6 a.m. are only 53% of the way toward optimality (Figure 3, blue bar). In particular, only 8% of our US students find work specifically at 6 a.m. to be optimal, the proportion rising slightly at 7 a.m. and rather more at 8 a.m. (dashed red line).

As the morning passes, the work day draws closer to optimality, reaching 70% at around 8 a.m. and 80% by 10 a.m. The highest approach to optimality for any single start times is around noon or 1 in the afternoon. Both achieve 81% optimality.

However, no single start time achieves as near an approach to optimality as would a mixed system in which students could start their 6-h class block at the beginning of their own personal optimum block of time—each student in effect looking through the whole day and choosing a block of 6 h that gives him or her the highest “feeling best” score. Hence, there would not be a single start time for the university, no “one size fits all” start, but rather a diversity of start times for a diversity of students. That system would bring us 88% of the way toward a fully optimal learning day; statistically it is significantly better (at *p* < 0.001) than any of the “one time fits all” starts. Of course, that is still not all the way to a fully optimal day which would be 100%, but it is far in that direction, and the chronotype-matched start is closer to optimal than any one of the fixed start times.

## Discussion

In line with neuroscience-based sleep research, survey-based data show that these undergraduates have a marked preference for much later working times than is now usual. Students' perceptions of their own optimal working times fit well with GBT waking at 9:27 a.m. (and hence starting the work day at 11:27) and sleeping at 1:27 a.m. Any work day in this range would reduce health risks and improve performance by reducing sleep deprivation and circadian disruption. For a single start time, the data suggests that starting anywhere between 11 a.m. and 1 p.m. would be close to optimal for these undergraduate students.

Analysis separately by chronotype shows that there is no single morning starting time that could be adopted without significantly disadvantaging some students. Early through mid-morning start times up to 10 or 11 disadvantage students with strong or even moderate evening preferences—about half of all students. These variations in biological timings are most acute for evening chronotypes as early starts are known to have a strong negative impact on health, mental health, and academic performance generally from early puberty onwards (Roenneberg et al., 2007; Preckel et al., 2013). SM data have a more direct focus on academic performance alone that show highly significant disadvantages of early starts for definitely evening types in morning hours (7 a.m. to noon) and excluding evening hours that are more likely to be optimal (7 p.m. to midnight).

Start times around noon or a couple of hours later are good for all. Such later start times appear likely to improve performance and to lower health risks, though practical issues and cost/benefit analysis require future research. SM and NM are scalable, flexible, and could be developed further in other contexts, for example matching chronotypes to shift work (Vetter et al., 2015), optimal performance at different ages, and different elements in performance such as working memory (Muto et al., 2016). An individual's *i*GBT may have applications in drug administration (Bass and Lazar, 2016), mental health (Wulff et al., 2010), and athletic performance. Previous research may need reassessment in light of differences between UTC and GBT.

However, no single start time achieves as near an approach to optimality as would a mixed system in which students could start their working day at the beginning of their personally optimal time. For a 6-h work day, this would get to 88 points out of 100. Longer periods of study, such as 8 h, could be accommodated with a working day of 11 a.m. to 7 p.m. This could create a time slot for early chronotypes before noon, and a time slot after 6 p.m. for later chronotypes (see also Beşoluk et al., 2011; Vetter et al., 2015).

There are limitations in this study including small sample size, and the focus on academic performance. The possible role of sleep hygiene—such as advice to stop using screen-based technologies in the last hour before sleep—can play a smaller, but significant role in reducing sleep deprivation, just as a healthy life style can impact on better health. The authors' awareness of these issues led to making the study easily replicable in other universities and other working contexts. Indeed, both the neuroscience-based, theoretical model, and the survey-based, empirical model were developed so that they could be applied to any individual, group, or age.

The findings here suggest that the Centers for Disease Control's recommendation of starts no earlier than 8:30 a.m. for high school students takes a step in the right direction but does not go far enough: 8:30 a.m. is still far earlier than the times indicated as optimal both here and in the wider sleep literature (see also Matchock and Mordkoff, 2009; Foster et al., 2013; Haraszti et al., 2014; Meltzer et al., 2014; van der Vinne et al., 2015).

Later starts in education have long been recommended and yet have not been implemented for a variety of reasons. The strength of evidence for change, and the lack of evidence for retaining current times, are now well-known. This study offers additional evidence for much later starts, and bringing universities into the discussion for later start times. The data suggest that aligning institutional schedules to individual's chronotypes to optimize academic performance and student health is a critical issue in improving higher education.

## Ethics statement

This study was performed in accordance with relevant institutional and national guidelines. The International Survey Center is a private, not-for-profit scientific charity founded in Australia in the 1980s and exempt from government institutional review. Confidentiality of survey respondents is strictly protected. Its 2014 Developmental Survey was publically available online when the US students filled it out. To provide relevant examples in statistics lectures, the students were invited to participate in the online developmental survey that the International Survey Center was running at the time. Survey-taking enhances student engagement as research has long shown that respondents enjoy well-designed surveys that they find relevant (Krosnick, 1991; Frymier and Shulman, 1995; Schaeffer and Presser, 2003). In alignment with ESRC (2015) Framework for Research Ethics standards (although not required here), in class, the professor provided information about the purpose, topics, methods, and potential uses of the survey; emphasized voluntary, confidential participation; and pointed out that students might enjoy taking the survey and comparing their responses to their peers'. Participation was not required. If students went to the survey and preferred not to continue they were free to decline to start or to opt out at any time; answering or skipping each question was voluntary (no questions required answers in order to proceed). The students were requested to mention their university in a source question, so that their responses could be filtered from others and provided to the faculty member. No identifying information was collected.

## Author contributions

Study design: ME; Implementation: ME and JK; Writing paper: ME, PK, and JK; Survey and Statistical analysis, JK and ME; Neuroscience analysis, PK.

## Funding

Science + Technology in Learning has funded this research.

### Conflict of interest statement

The authors declare that the research was conducted in the absence of any commercial or financial relationships that could be construed as a potential conflict of interest.
