# Supplementary material for: Identifying the Best Times for Cognitive Functioning Using New Methods: Matching University Times to Undergraduate Chronotypes
Source: Front Hum Neurosci. 2017 Apr 19;11:188. doi: 10.3389/fnhum.2017.00188 (PMC5395635; doi:10.3389/fnhum.2017.00188)
Supplement: Supplementary file 1 [file DataSheet1.docx]

**Technical Appendix**

*1. Raw data: Frequencies for selected questions*

**Appendix Table A. Answers to selected questions "Do you usually feel at your best at these times...". US university students.**

**-------------------------------------**

**Time**

**-----------------------**

**Mid-**

**Answer Score 6am 10am 4pm night**

**------------+------------------------**

**Yes!! 100 | 2 21 28 13**

**Yes 75 | 6 38 42 14**

**?? 50 | 14 23 18 29**

**No 25 | 30 11 10 32**

**No!! 0 | 49 8 2 11**

**------------+------------------------**

**Total 100% 100% 100% 100%**

**Mean 21 63 71 47**

**Std. dev. 25 28 25 30**

**Cases 189 194 191 188**

**-------------------------------------**

*2. Equivalent questions "how awake and alert..."*

Our central "do you usually feel at your best..." questions seem to measure much the same "optimal time of day concept" as an alternative series of questions: "How awake and alert do you feel... Very alert!/ Alert/ Somewhat alert/ In between/ Somewhat sleepy/ Sleepy/ Very sleepy!".

Answers to the two questions show the same time patterns (Appendix, Table B) and are highly correlated (r=.58 on average).

**Appendix Table B. An alternative measure leads to the same conclusions. Question: "How awake and alert do you feel... Very alert!/ Alert/ Somewhat alert/ In between/ Somewhat sleepy/ Sleepy/ Very sleepy!" Scored in equal intervals between "Very alert"=100 and "Very sleepy"=0. Means, standard deviations, and correlations with the "feel your best" question. 9 to 5 workday highlighted. US university students.**

**---------------------------------------------------**

**Correlation with**

**"feel at your best"**

**Time Mean Std.Dev. for same hour Cases**

**-------+-------------------------------------------**

**5am | 17.0 27.2 .75 197**

**6am | 19.2 26.6 .80 197**

**7am | 27.1 29.4 .76 197**

**8am | 41.3 30.1 .71 194**

**9am | 57.0 29.7 .65 195**

**10am | 70.3 26.4 .61 196**

**11am | 79.2 22.6 .55 196 12noon | 83.5 20.9 .45 194**

**1pm | 84.9 19.5 .45 197**

**2pm | 83.6 21.3 .55 197**

**3pm | 82.2 21.0 .53 197**

**4pm | 80.7 21.6 .57 196**

**5pm | 80.6 21.0 .61 195**

**6pm | 80.2 18.8 .52 194**

**7pm | 79.7 18.5 .45 194**

**8pm | 76.4 20.3 .52 195**

**9pm | 70.6 23.8 .47 194**

**10pm | 62.6 27.3 .45 193**

**11pm | 49.1 30.5 .54 195**

**-------+-------------------------------------------**

The two questions could reasonably be taken as alternative measures of the same underlying concept: The correlations are in the moderate to high range, they have good face validity, and the alpha reliabilities are all in the acceptable range for 2-item scales (Eisinga, te Grotenhuis, and Pelzer 2012). Alpha reliability (for selected hours) are:

- for 6 a.m.: Alpha= .88
- for 9 a.m.: Alpha= .78
- for 12 noon: Alpha= .58
- for 3 p.m.: Alpha= .62
- for 6p.m. : Alpha= .67
- for 9p.m.: Alpha= .65

Combining them hour-by-hour into single "optimal time of day" measures would give a series of good two-item scales.

*3. Correlations, structure*

Optimality ratings for adjacent time periods are closely correlated – for example, people who like 6 in the morning are inclined to like 7 and 8 in the morning as well

**Appendix Table C. Correlations among answers to questions "Do you usually feel at your best at these times..." for selected times, 6a.m. to 10p.m. To keep the correlation matrix a reasonable size, only selected hours are shown. N=184 cases with complete information on all questions. US university students. Correlations over .40 are highlighted.**

**----------------------------------------------------------------------------------------------**

**_6am _8am _10am _noon _2pm _4pm _6pm _8pm _10pm**

**-------------+--------------------------------------------------------------------------------**

**_6am | 1.0000**

**_8am | 0.6664 1.0000**

**_10am | 0.2131 0.4941 1.0000**

**_noon | 0.0128 0.0582 0.5428 1.0000**

**_2pm | -0.1501 -0.1806 0.1960 0.6155 1.0000**

**_4pm | -0.1059 -0.0769 0.1336 0.4589 0.7391 1.0000**

**_6pm | -0.1481 -0.1425 -0.0045 0.2676 0.4942 0.7236 1.0000**

**_8pm | -0.2349 -0.2049 -0.0467 0.1232 0.2420 0.3281 0.6136 1.0000**

**_10pm | -0.1644 -0.2158 -0.1506 -0.0381 0.1198 0.1751 0.3793 0.6330 1.0000**

**_12mid | -0.0793 -0.1792 -0.2729 -0.2698 -0.0205 0.0593 0.1723 0.2915 0.6387**

**----------------------------------------------------------------------------------------------**

Broadly speaking there seem to be three periods in the main part of the day (leaving out late night sleeping hours and the earliest few hours of the morning). Factor analysis shows this clearly (Appendix Table D). The periods, familiar ones, are:

- Morning, roughly 8 a.m. to 11 a.m. (Factor 3), with noon overlapping this and the next period;
- Afternoon, roughly 1 p.m. to 5 p.m. (Factor 1), with 6p.m. overlapping this and the next period;
- Evening, roughly 7 p.m. to 10 p.m. or possibly later.

Within each of these periods people tend to have reasonably consistent feelings, but between them not so much so.

**Appendix Table D. Factor analysis of questions "Do you usually feel at your best at these times..." Factor loadings, oblimin rotation. N-180 US university students with complete information on all questions. Loadings over .50 are highlighted.**

**--------------------------------------------------**

**Time Factor1 Factor2 Factor3**

**--------------------------------------------------**

**8am | -0.1878 -0.1165 0.6761**

**9am | -0.0487 -0.1014 0.8576**

**10am | 0.1906 -0.0798 0.8650**

**11am | 0.3822 -0.0402 0.7947**

**12pm | 0.6564 -0.0370 0.5002**

**1pm | 0.7981 0.0178 0.2556**

**2pm | 0.9022 0.0703 0.0575**

**3pm | 0.8726 0.1084 -0.0694**

**4pm | 0.8285 0.2756 0.0298**

**5pm | 0.6922 0.4808 0.0039**

**6pm | 0.5785 0.6461 -0.0444**

**7pm | 0.2973 0.7665 -0.0458**

**8pm | 0.1645 0.9070 -0.0373**

**9pm | 0.0980 0.8825 -0.0866**

**10pm | 0.0289 0.7157 -0.1589**

**--------------------------------------------------**

**Eigenvalues for unrotated solution: 5.67, 3.57, and 1.66; all others under 1.0**

These are closely linked to self-ascribed morning chronotypes, with those who like the morning hours strongly identifying as morning chronotypes and those who dislike evening hours slightly less likely to (Table E, column 1). Those who like afternoon hours are not distinctive.

**Appendix Table E. Links between questions "Do you usually feel at your best at these times..." with answers grouped as implied by the factor analysis in Appendix Table B, on the one hand, and self-assessed chronotype, on the other. OLS regression estimates (probit regressions show the same pattern albeit in a less intuitive form). US university students.**

**----------------------------------------------------------------------------**

**Self-assessed chronotype**

**--------------------------------------------------------------**

**Definitely**

**Morning In between Evening evening**

**Feel best (1) (2) (3) (4)**

**----------------------------------------------------------------------------**

**8am to 11am 0.567*** 0.027 -0.208** -0.383*****

**1pm to 5pm -0.099 0.081 0.056 -0.037**

**7pm to 10pm -0.173** -0.096 0.003 0.264*****

**male 0.001 0.165* -0.106 -0.056**

**----------------------------------------------------------------------------**

**R-sq 0.389 0.035 0.052 0.247**

**N 178 178 178 178**

**----------------------------------------------------------------------------**

**Standardized beta coefficients**

*** p<0.05, ** p<0.01, *** p<0.001**

A similar pattern holds in reverse for those who self-identify as "definitely evening" chronotypes (column 4). Those who like morning hours fairly strongly reject the "definitely evening" chronotype while those who like the evening hours tend to accept it – but not all that strongly. Interestingly, it is views about the *morning* hours that mostly determine acceptance or rejection of a "definitely evening" chronotype.

The chronotypes between morning and "definitely" evening are not strongly linked to time preferences (columns 2 and 3).

*4. Theoretical model*

Determining the starting times is a mixture of biologically determined and socially decided periods of time, and this can be arranged within the 24-hour day in a variety of different ways. The theoretical model is a close match with the SM in indicating that 1 p.m. is the beginning of a period best suited for all students’ cognitive performance.

Broadly the movement of starting times to 11a.m. makes a critical difference to most students. The three periods set out in Table C of Morning (8 a.m. to 11 a.m.); Afternoon (1 p.m. to 5 p.m.) and Evening (7 p.m. to 10 p.m.) are reflected in the NM model. The gap between morning and afternoon in Table C data (between 11 a.m. and 1 p.m.) indicates the best starting times period with 1 p.m. clearly better for evening types. The Evening period in Table C indicates the best ending times (7 p.m. to 10 p.m.) in the NM model, and the gap between the end of evening and of end of morning (10 p.m. to 11 a.m.) gives a better sleep window for ‘definitely evening’ chronotypes.

The NM model and GBT here also considered specific cognitive functions (Diekelmann and Born), 2010), developmental cortical changes (Schmitt et al., 2014), twilight patterns (Welbergen, 2008), and societal attitudes to timing changes (Roenneberg, 2012).

**References**

Eisinga, R., te Grotenhuis, M., and Pelze, B. (2013). The reliability of a two-item scale. Int. J. Public Health 58, 637–642. doi: 10.1007/s00038-012-0416-3 Diekelmann, S., and Born, J. (2010). The memory function of sleep. Nat. Rev. Neurosci. 2, 114–126. doi: 10.1038/nrn2762

Roenneberg, T. (2012). Internal Time. Cambridge; London: Harvard University Press.

Schmitt, J. E., Neale, M. C., Fassassi, B., Perez, J., Lenroot, R. K., Wells, E.M., et al. (2014). The dynamic role of genetics on cortical patterning during childhood and adolescence. Proc. Natl. Acad. Sci. U.S.A. 111, 6774–6779.doi: 10.1073/pnas.1311630111

Tassi, P., and Muzet, A. (2000). Sleep inertia. Sleep Med. Rev. 4, 341–353. doi: 10.1053/smrv.2000.0098

Welbergen, J. A. (2008). Variation in twilight predicts the duration of the evening emergence of fruit bats from a mixed-species roost. Anim. Behav. 75, 1543–1550. doi: 10.1016/j.anbehav.2007.10.007
